# Empirical factors affecting memory in collaborative versus nominal groups

**DOI:** 10.3389/fpsyg.2023.1214910

**Published:** 2024-01-08

**Authors:** Melissa J. Guynn

**Affiliations:** Department of Psychology, New Mexico State University, Las Cruces, NM, United States

**Keywords:** collective memory, collaborative memory, collaborative inhibition, retrieval strategy disruption, nominal group, collaborative group, group remembering, cross cuing/cueing

## Abstract

When individuals collaborate to try to retrieve some encoded information, not surprisingly, the collaborative group typically remembers more than does any individual. When the non-redundant output from the individuals is combined, however, this nominal group often, surprisingly, remembers more than does the collaborative group. This finding is known as *collaborative inhibition*. The finding of collaborative inhibition, that collaborative groups remember less would be predicted given the summed non-redundant memories of an equal number of individuals remembering alone, indicates that there is something about remembering in a collaborative group that impairs the performance of the individuals in that group. Research directed toward what that something is has focused on both social and cognitive factors, with the consensus being that cognitive factors play the more important role. An extensive body of work on this topic has accumulated over the past 25+ years, with researchers proposing theoretical explanations and generating empirical data revealing the conditions under which this collaborative inhibition is more versus less likely to occur. The purpose of this review is to summarize those empirical factors to provide a resource for researchers interested in pursuing this work.

## Introduction

It is a conventional notion that collaborating on some task with others generally produces a more desirable outcome than does working alone. This sentiment is exemplified in numerous quotes by notable individuals:

“It is the long history of humankind…that those who learned to collaborate… most effectively have prevailed” (Charles Darwin).

“Alone we can do so little; together we can do so much” (Helen Keller).

“Teamwork makes the dream work” (John C. Maxwell).

These adages (and many others) express the common sentiment that “two heads are better than one” (C. S. Lewis). Many accept them as truth, but a skeptic or a curious behavioral researcher might ask for the evidence for such claims or whether there might be some exceptions to them. Although a complete exploration of this issue is beyond the scope of this paper, one relevant line of work will be reviewed.

Weldon and Bellinger (1997) were interested in this topic as it pertains to human memory. They did not question the result that groups remember more than do individuals on average (Clark and Stephenson, 1995) but they did wonder about the effect of the group on the individual and whether collaborative groups maximized possible memory performance. In their review, they noted a finding from the brainstorming literature (Bouchard and Hare, 1970; Diehl and Stroebe, 1987, 1991), that individuals working together to brainstorm solutions to problems typically produce fewer novel solutions than do an equal number of individuals working alone, and they wondered whether this finding extended to human memory. To explore this, participants studied word lists or the *War of the Ghosts* folktale. During the test, participants either worked alone or collaborated with two others to try to remember. Not surprisingly, collaborative groups remembered more on average than did individuals. But the critical comparison was between collaborative groups and *nominal* groups, which were formed by pooling the non-redundant responses of an equal number of individuals who worked alone. The result replicated that from the brainstorming literature; collaborative groups remembered less on average than did nominal groups. Something about collaborating with others to remember hurt memory performance, and Weldon and Bellinger termed this finding *collaborative inhibition*.

A hypothetical example may help to illustrate the pattern (see Table 1). Imagine that participants are asked to remember either collaboratively in groups of three or individually and then combined into nominal groups of three. In the table, different uppercase letters of the alphabet are used to depict unique stimuli (e.g., words) remembered at test. Individual memory, or the average of what individuals remember on their own (i.e., 4), is worse than collaborative group memory, or the total of what the collaborative group members remember together (i.e., either 9 or 15, depending on the scenario depicted). And in the scenario showing *collaborative inhibition*, collaborative group memory (i.e., 9) is worse than nominal group memory (i.e., 12), or the total of what the individuals remember on their own, excluding any redundancies. Another scenario shows *collaborative facilitation*, which is the finding that collaborative group memory (i.e., 15) is better than nominal group memory (i.e., 12). This pattern has not generally been found but could occur if collaborative group members cross cue each other, such that what one person remembers reminds a fellow group member of something they might not have otherwise remembered (Meudell et al., 1992, 1995).

**Table 1:** Hypothetical example of individual versus nominal group memory and two possibilities for collaborative group memory.

	Remember individually	Remember collaboratively with inhibition	Remember collaboratively with facilitation
Participant 1	A B C	A B	A B C D
Participant 2	E F G H	E F G	E F G H I
Participant 3	J K L M N	J K L M	J K L M N O

Individual memory = (3 + 4 + 5)/3 = 4
Nominal group memory = 3 + 4 + 5 = 12
Collaborative group memory with inhibition = 2 + 3 + 4 = 9
Collaborative group memory with facilitation = 4 + 5 + 6 = 15

The unique uppercase letters represent unique studied items remembered by participants.

Weldon and Bellinger (1997) proposed both social and cognitive factors that could produce the effect, but subsequent work (Weldon et al., 2000) suggested that the mechanism was more likely cognitive (e.g., retrieval disruption; see also Basden et al., 1997) than social (e.g., social loafing; but see Ekeocha, 2021 for evidence suggesting that social factors should not yet be ruled out). Possible cognitive explanations include production blocking, retrieval inhibition, retrieval blocking, and retrieval strategy disruption. Production blocking is the idea people forget some things they otherwise would have remembered because of the need to wait their turn while fellow group members share what they remember (Diehl and Stroebe, 1987; Andersson et al., 2006; *cf.* Wright and Klumpp, 2004; Hyman et al., 2013). Retrieval inhibition is the idea that information remembered by fellow group members permanently suppresses some memory representations of others in the collaborative group (Barber et al., 2015). Retrieval blocking is the idea that information remembered by fellow group members temporarily restricts access to some memory representations of others in the collaborative group (Barber et al., 2015). Retrieval strategy disruption is the idea that people have a preferred strategy they use to guide their retrieval, and information remembered by fellow group members disrupts this preferred retrieval strategy and thereby hurts memory (Basden et al., 1997; Finlay et al., 2000; Marion and Thorley, 2016). This last account is the one that has received the most attention and support thus far.

Other research has explored the effect of empirical factors and revealed conditions under which collaborative inhibition may be reduced or eliminated. This research has accordingly revealed conditions under which collaborative inhibition may be increased. Although some of this empirical work has been conducted to evaluate theoretical explanations, the purpose of this review is not the theoretical implications *per se*. The interest in this paper is the empirical factors, because in order to evaluate theoretical explanations of any effect, it is generally important for the effect to occur. In other words, although the combined empirical/theoretical work has generally supported the retrieval strategy disruption hypothesis, other theoretical explanations have not been ruled out. One goal of this review is to identify the empirical factors that increase collaborative inhibition, to facilitate further evaluation of these alternate theoretical explanations of the effect.

Also, just because memory in a collaborative group is worse than that in a nominal group, this does not necessarily mean that there are only costs from collaborating. There could be benefits as well. Specifically, Meudell et al. (1992, 1995) proposed that individuals working together might cross cue each other, such that what one person remembers reminds a fellow group member of something they might not have otherwise remembered. In five experiments, participants did not remember more new studied items (on a second test) when working with another participant than when working alone. This result does not necessarily mean that cross cueing did not occur, but if it did occur, any beneficial effect was offset by a detrimental one. Meudell et al. concluded that there is likely an unidentified mechanism (e.g., that which produces collaborative inhibition) that impairs memory when people collaborate to remember. Thus, another goal of this review is to identify the empirical factors that decrease collaborative inhibition, to facilitate further evaluation of the possibility of cross cueing among collaborative group participants leading to collaborative facilitation.

The variables impacting the extent of any collaborative inhibition are organized in terms of a tetrahedral model of memory experiments (Jenkins, 1979), according to which, memory researchers should consider four types of variables in their studies: (1) research participants, (2) stimulus materials, (3) the encoding conditions, and (4) the retrieval conditions. Of course, the effect of any manipulated variable may also depend on other variables either manipulated or held constant. Some of this work was intended to evaluate theoretical explanations of the effect. In each case, where appropriate, the theoretical relevance of any particular result will be included. Each of these factors that affect the extent of collaborative inhibition could be considered a cognitive factor.

## Participants

Two somewhat different types of participant factors have been found to affect collaborative inhibition. One pertains to the cognitive capacity of the participants themselves, whereas the other is simply the number of participants in the collaborative group. A third participant factor, the level of acquaintance of the participants in the collaborative group, has been studied but has not produced consistent results, as also mentioned below.

Variables such as working memory capacity, executive function, and attentional capacity can be related to the extent of collaborative inhibition, but the nature of their effect seems complex, as evidenced by the results of three studies. In one such study, Hood et al. (2023) found that collaborative inhibition was greater for participants with lower working memory capacity than for participants with higher working memory capacity (as measured by operation, reading, and symmetry span tasks). Hood et al. suggested that participants with lower working memory capacity are less able (compared to participants with higher working memory capacity) to maintain retrieved items in mind when distracted by fellow collaborative group members. Conversely, Barber and Rajaram (2011) found that participants who performed an executive depletion task before taking their memory test experienced no more collaborative inhibition than participants who did not perform such a task. The reason for these discrepant results is unclear. It may be that the executive depletion task did not functionally reduce the working memory capacity of the participants for the subsequent retrieval phase (Barber and Rajaram, 2011), or it may be that the relationship between working memory capacity and collaborative inhibition occurs because of an effect at encoding rather than at retrieval (Hood et al., 2023).

Consistent with an encoding interpretation, a study by Pereira-Pasarin and Rajaram (2011) found that dividing attention at encoding eliminated collaborative inhibition. They suggested that this could occur if attention at encoding is useful for detecting relationships among studied stimuli that could later guide retrieval at test. Participants who encode more relational information (i.e., full attention condition) thus could use more relational information to guide retrieval and thus would be more disrupted by collaboration. Participants who encode less relational information (i.e., divided attention condition) thus would use less relational information to guide retrieval and thus would be less disrupted by collaboration. Further support for this interpretation came from finding that dividing attention at encoding decreased organization at the time of retrieval as measured by adjusted ratio of clustering (ARC) scores (Roenker et al., 1971) computed on the recall output.

Despite the plausible nature of these explanations, the fact that two similar variables appear to affect the extent of collaborative inhibition in different ways has yet to be explained. Why is it that the extent of collaborative inhibition is increased for participants with low working memory capacity but decreased for participants who encode under conditions of divided attention? It is not clear at this point whether factors such as working memory capacity, executive function, and attentional capacity should be considered functionally equivalent or not, nor whether an effect of such variables is because of processes happening at encoding, at retrieval, or at both.

The size of the collaborative group also generally impacts the extent of the collaborative inhibition, with a greater deficit found with larger groups (Basden et al., 2000; Marion and Thorley, 2016). Studies have tended to find collaborative inhibition reliably in groups of three or four participants (Basden et al., 1997; Weldon and Bellinger, 1997; Basden et al., 2000) and sometimes but not always in groups of two (Basden et al., 2000; *cf.* Wright and Klumpp, 2004). This finding is consistent with all of the cognitive explanations of collaborative inhibition (i.e., production blocking, retrieval inhibition, retrieval blocking, and retrieval strategy disruption). This is because in each case, it is the presence of fellow group members that causes the impairment. A greater number of group members would be expected to produce greater impairment.

Another factor that was expected to affect the extent of collaborative inhibition is the level of acquaintance among the participants. The idea is that if individuals know each other well, each may be able to predict what the other is thinking to some extent and thus they may communicate more effectively (i.e., and interfere with each other to a lesser extent) compared to individuals who do not know each other well or at all. The results with regard to this factor are mixed. Although Andersson and Rönnberg (1995, 1996) found less productivity loss (e.g., collaborative inhibition) in dyads comprising friends than non-friends, others have not replicated this effect (Johansson et al., 2000). Equivalent collaborative inhibition has been demonstrated in groups of friends and non-friends (Peker and Tekcan, 2009; Harris et al., 2013) and in married couples for both non-personal and personal information (Ross et al., 2004; Harris et al., 2017). Thus, the level of acquaintance is not a variable that consistently affects the level of any collaborative inhibition.

## Stimulus materials

A stimulus factor that can impact collaborative inhibition is the number of conceptual categories in the study list and the size of those categories (i.e., the number of exemplars per category), and the nature of the impact depends on the dependent measure. Basden et al. (1997, Experiment 1) found that when number of categories recalled was considered, collaborative inhibition occurred when there was a large number of small categories (i.e., 6 exemplars in each of 15 categories), but not when there was a small number of large categories (i.e., 15 exemplars in each of 6 categories). When the number of instances recalled per category was considered, the opposite result was found. The idea is that larger sets of stimuli permit more opportunities for idiosyncratic organization, which can be disrupted by collaboration, consistent with the retrieval strategy disruption hypothesis.

Work by Andersson and Rönnberg (1995) also revealed some effects of stimulus materials on the extent of collaborative inhibition. In one study, collaborative inhibition was greater when participants tried to remember unrelated words than when they tried to remember a story and answer questions about it (although there was a confounding, in that memory for the unrelated words was tested via free recall and memory for the story was tested via cued recall, and test type has also been found to affect collaborative inhibition). In another study, participants answered questions about an instructional videotape. Some questions required that details be remembered (i.e., elaboration not required) and others required reasoning in order to be answered correctly (i.e., elaboration required). Collaborative inhibition was greater when the questions did not require elaboration. Andersson and Rönnberg suggested that collaborative inhibition was reduced when the task was more complex, but another possibility is that the less complex stimulus materials were more susceptible to being organized in idiosyncratic ways across participants and thus more susceptible to disruption from collaboration.

In a meta-analysis with stimulus type as one of the moderator variables, Marion and Thorley (2016) found partial support for the prediction of greater collaborative inhibition when the stimulus materials are unrelated or uncategorized (e.g., lists of unrelated words) than when they are related or can be categorized in some way (e.g., lists of words that belong to the same categories, sentences that make up a meaningful story). Specifically, they found somewhat greater collaborative inhibition for word lists than for story materials, consistent with Andersson and Rönnberg (1995), but no significant difference between unrelated and related word lists. This finding is not necessarily inconsistent with Basden et al. (1997, Experiment 1), as lists of related words do permit idiosyncratic organization that can be disrupted by collaboration.

## Encoding conditions

Another factor that can impact collaborative inhibition is the extent to which participants have a similar experience at encoding. Shared encoding can eliminate collaborative inhibition (*cf.* Andersson and Rönnberg, 1995; Finlay et al., 2000; Barber et al., 2012; Harris et al., 2013). With individual encoding, encoding the study list items in the same order as fellow collaborative group participants, as opposed to in different orders, can reduce collaborative inhibition (Finlay et al., 2000). Relatedly, encoding and retrieving using the method of loci (vs. using whatever strategy a participant thought to use) eliminated collaborative inhibition in a serial recall task (Saraiva et al., 2016). This effect could have occurred because of a shared strategy at encoding, at retrieval, or at both.

Factors that lead to better encoding of the relationships among or the organization of the stimulus materials can also decrease collaborative inhibition. Basden et al. (2000) found that the collaborative inhibition that occurred after a single study and test was eliminated after repeated study and test. Pereira-Pasarin and Rajaram (2011) found that collaborative inhibition and organization at the time of retrieval as evidenced by adjusted ratio of clustering (ARC) scores (Roenker et al., 1971) were decreased by repeating the stimulus materials three times at study compared to just once. Reysen et al. (2018) found that survival processing at encoding eliminated collaborative inhibition but there is not yet a convincing explanation of this effect.

The length of the delay between study and test has been found to impact collaborative inhibition. Takahashi and Saito (2004) found collaborative inhibition following incidental learning of story material (i.e., a fairy tale) when the test immediately followed the story but not when the test occurred 1 week later. Congleton and Rajaram (2011) found collaborative inhibition following intentional learning of categorized words when the test occurred at a short delay of 7 min but not at a long delay of 2 h. Abel and Bäuml (2017) found collaborative inhibition following intentional learning of uncategorized words when the test occurred at a short delay of 5 min but not at a long delay of 24 h.

This result can be explained if test delay disrupts the encoded organization of the stimulus materials (Takahashi and Saito, 2004) or the access to the study context (Abel and Bäuml, 2017), such that any idiosyncratic organizational retrieval strategy is reduced on a delayed test compared to an immediate test. Thus, if collaboration disrupts idiosyncratic organizational retrieval strategies, then there is less to disrupt on a delayed test than on an immediate test, and consequently, any performance advantage for nominal groups over collaborative groups should diminish on a delayed test compared to an immediate test. This allows the possibility that what fellow collaborative group members remember on a delayed test could actually help fellow group members to remember things they might not have otherwise remembered (i.e., via cross cueing) rather than hurting fellow group members via retrieval strategy disruption (Takahashi and Saito, 2004; Congleton and Rajaram, 2011).

## Retrieval conditions

A variety of manipulations at the time of retrieval can impact the extent of collaborative inhibition. Many (if not most) of these seem to affect the organizational strategies that participants are able to use at the time of retrieval. Collaborative inhibition is generally greater when participants are less constrained (and thus more free to use their own preferred organizational strategies) in the order in which they output remembered information. This is because these preferred organizational strategies are disrupted by fellow collaborators, consistent with the retrieval strategy disruption hypothesis. For example, Congleton and Rajaram (2011) found that collaborative inhibition disappeared after repeated testing, which likely reinforced the organization of the stimuli that was used to guide retrieval.

Basden et al. (1997, Experiment 2) did not find collaborative inhibition for a categorized list of words on a free recall test (they shared a plausible explanation for this unexpected result), but they did find it when the category labels were available as retrieval cues for participants throughout the recall test. The presence of the category label cues likely permitted more frequent switching among categories at test and thereby disrupted the use of organization of the stimuli to guide retrieval. A somewhat related factor for uncategorized lists of paired associates is whether a separate retrieval cue (the cue word in a studied cue-target pair) is provided to participants for each target at recall (i.e., whether recall is free vs. cued by paired associates). Finlay et al. (2000) found collaborative inhibition when the test was free recall of the pairs but not when the test was cued recall of the targets given the cues. The presence of the cues likely prevented participants from using the organization of the stimuli to guide retrieval.

Another factor for a categorized word list is whether recall must be completed for any given category before exemplars from a different category may be recalled (i.e., whether participants are forced to cluster their recall by category). Basden et al. (1997) did not find collaborative inhibition when participants were forced to cluster their recall by category, thus obviating the need for their own organizational strategies (Experiment 4), but they did find collaborative inhibition when participants were not so constrained, thus necessitating that participants rely on their own organizational strategies, which were then disrupted by collaboration (Experiment 3, whole-list recall). In contrast, Meade and Roediger (2009) did obtain collaborative inhibition on a cued recall test when participants were forced to cluster their recall by category, but they speculated that their results differed because Basden et al. used low frequency category exemplars whereas Meade and Roediger used high frequency category exemplars.

Another factor is whether participants each try to recall the same study list items (i.e., participants tried to free recall the entire list) versus different study list items (i.e., participants were cued with category labels at test to recall subsets of the list). Basden et al. (1997, Experiment 3) did not obtain collaborative inhibition when participants each tried to recall a different subset of the study list items but they did when all participants tried to recall the entire study list. This result again likely occurred because participants relied on preferred organizational retrieval strategies to a greater extent on free recall than on cued recall, and these preferred organizational retrieval strategies were disrupted by collaboration.

An interesting experiment by Wright and Klumpp (2004) provided further evidence that it is the content remembered by fellow collaborative group members that causes the impairment. Wright and Klumpp (2004) compared performance in a nominal group to that in a standard collaborative group, whereby participants took turns recalling study list items and heard the items recalled by another participant, and to a modified collaborative group, whereby participants took turns recalling study list items but did not hear the items recalled by another participant. Collaborative inhibition occurred in the former condition but not the latter, prompting the conclusion that it is the product (i.e., what others remember) and not the process (i.e., turn taking) of collaboration that hurts memory and thus supports retrieval strategy disruption and not production blocking as the mechanism that causes collaborative inhibition.

Most research has focused on tests of explicit episodic memory, but studies that have included tests of implicit memory or semantic memory have not found evidence of collaborative inhibition. Consistent with the retrieval strategy disruption hypothesis, collaborative inhibition has generally not been found on memory tests for which using a preferred retrieval strategy would not be useful. Rossi-Arnaud et al. (2017) found that the amount of priming on implicit tests of word fragment completion and category exemplar generation was statistically equivalent for collaborative and nominal groups. Andersson and Rönnberg (1996) found collaborative inhibition in an explicit task involving remembering dot patterns but not in a comparable implicit task involving dot pattern completion. They also found collaborative inhibition when participants had remember a story (i.e., episodic memory) but not when participants had to answer an equal number of general knowledge questions within the story domain (i.e., semantic memory). Within the explicit episodic domain, Saraiva et al. (2023) found less collaborative inhibition in serial recall than in free recall, and moreover, when participants were required to take turns, the collaborative inhibition was eliminated in serial recall and reduced in free recall.

## Discussion

The purpose of this review is to provide a guide for researchers interested in collaborative memory, including collaborative inhibition and the possibility of collaborative facilitation. When evaluating theoretical explanations of collaborative inhibition, one might wish to ensure there is a difference between nominal and collaborative groups. When exploring the conditions under which collaborative facilitation might occur, one might wish to minimize or eliminate collaborative inhibition so that any effect of cross cueing can be evidenced. This review considered characteristics of participants, stimulus materials, encoding conditions, and retrieval conditions (Jenkins, 1979). Regarding participants, collaborative inhibition tends to be greater when there are more participants in a group and when participants have lower working memory capacity. Regarding stimulus materials, collaborative inhibition tends to be greater when there is a greater number of different ways the stimuli can be organized (e.g., idiosyncratically by participants). Regarding encoding conditions (including the delay between encoding and retrieval), collaborative inhibition tends to be increased to the extent that participants have different experiences at encoding and decreased to the extent that stimulus materials are more organized at encoding. Regarding retrieval conditions, collaborative inhibition tends to be increased when the output order is less constrained and decreased when it is more constrained.

The effects of these factors are often consistent with the retrieval strategy disruption explanation of collaborative inhibition. Although this is the account that has received the most attention and support thus far, others have not necessarily been ruled out (i.e., retrieval blocking) and have even received some empirical support (i.e., retrieval inhibition; Barber et al., 2015). A challenge for researchers is to design experiments to evaluate these alternate explanations. In these circumstances, researchers likely want to produce or maximize collaborative inhibition (because it seems easier to study an effect that actually occurs). Accordingly, researchers with such an interest might want to implement conditions such as those listed in the top half of Table 2.

**Table 2:** Factors affecting the extent of collaborative inhibition.

Factors likely to increase collaborative inhibition
*Participants*
Larger group size (e.g., more than three participants per group)
Participants with lower working memory capacity
*Stimulus materials*
Larger stimulus sets (e.g., larger number of exemplars per conceptual category)
Word lists (e.g., as opposed to stories)
Lists of unrelated (e.g., as opposed to related) words
*Encoding conditions*
Participants work alone (e.g., vs. with other participants) to encode
Different participants encode stimuli in different orders
One study session and one test session
Short or no delay between study and test
*Retrieval conditions*
Free recall (e.g., as opposed to paired associate cued recall)
Free recall with all category labels available to be used as retrieval cues
Participants try to recall the same stimuli as other participants
Participants hear stimuli recalled by other participants
Explicit memory test
Free responding (i.e., not enforcing turn taking)
Factors likely to decrease collaborative inhibition
*Participants*
Smaller group size (e.g., two participants per group)
*Stimulus materials*
Smaller stimulus sets (e.g., smaller number of exemplars per conceptual category)
Stories (e.g., as opposed to word lists)
Lists of related (e.g., as opposed to unrelated) words
*Encoding conditions*
Divided attention at encoding (discussed in the Participants subsection)
Participants work with other participants (e.g., vs. alone) to encode
All participants encode stimuli in the same order
Multiple study and/or test sessions
Long delay between study and test
*Retrieval conditions*
Paired associate cued recall (e.g., as opposed to free recall)
Participants forced to recall category by category (especially with low frequency category exemplars)
Different participants try to recall different stimuli
Participants do not hear stimuli recalled by other participants
Forced turn taking in free recall
Implicit memory test
Semantic memory test
Serial recall test

An empirical issue that pertains to the real-world applicability of this research is the distinction between factors that increase collaborative inhibition by helping the performance of individuals (and nominal groups) and those that increase collaborative inhibition by hurting the performance of collaborative groups. Although both have relevance for testing theoretical accounts, the latter would seem to have greater applicability to remembering in the real world.

The focus of this review has been on how working in a collaborative group negatively impacts individual performance. But other research not reviewed here has explored how working in a collaborative group might also positively impact individual performance subsequent to the collaboration. Working in a collaborative group may provide an opportunity to restudy material that may not have been remembered during collaboration but that can then be remembered on a later individual memory test.

But if participants can cross cue each other, such that they each remember some things they would not have otherwise remembered, then working in a collaborative group could actually positively impact individual performance on the collaborative memory test itself. In these circumstances, researchers likely want to reduce or eliminate collaborative inhibition (so that any effect of cross cueing could be more easily observed). Accordingly, researchers with such an interest might want to implement conditions such as those listed in the bottom half of Table 2.

This research domain can be considered an exploration of the effect on the memory of an individual as a result of working in a group. Alternatively, it can be considered an exploration of whether remembering is optimized in a collaborative group. In this sense, perhaps a better quote to reflect this idea is one by Gestalt psychologist Kurt Koffka who said, “It has been said the whole is more than the sum of its parts. It is more correct to say that the whole is something else than the sum of its parts.”

## Author contributions

The author confirms being the sole contributor of this work and has approved it for publication.

## References

[ref1] Abel M. Bäuml K.-H. T. (2017). Collaborative remembering revisited: study context access modulates collaborative inhibition and later benefits for individual memory. Mem. Cogn. 45, 1319–1334. doi: 10.3758/s13421-017-0737-9, PMID: 28770540

[ref2] Andersson J. Hitch G. Meudell P. (2006). Effects of the timing and identity of retrieval cues in individual recall: an attempt to mimic cross-cueing in collaborative recall. Memory 14, 94–103. doi: 10.1080/09658210444000557, PMID: 16423746

[ref3] Andersson J. Rönnberg J. (1995). Recall suffers from collaboration: joint recall effects of friendship and task complexity. Appl. Cogn. Psychol. 9, 199–211. doi: 10.1002/acp.2350090303

[ref4] Andersson J. Rönnberg J. (1996). Collaboration and memory: effects of dyadic retrieval on different memory tasks. Appl. Cogn. Psychol. 10, 171–181. doi: 10.1002/(sici)1099-0720(199604)10:2<171::aid-acp385>3.0.co;2-d

[ref5] Barber S. J. Harris C. B. Rajaram S. (2015). Why two heads apart are better than two heads together: multiple mechanisms underlie the collaborative inhibition effect in memory. J. Exp. Psychol. Learn. Mem. Cogn. 41, 559–566. doi: 10.1037/xlm0000037, PMID: 25068855 PMC4309738

[ref6] Barber S. J. Rajaram S. (2011). Collaborative memory and part-set cueing impairments: the role of executive depletion in modulating retrieval disruption. Memory 19, 378–397. doi: 10.1080/09658211.2011.575787, PMID: 21678155 PMC3117231

[ref7] Barber S. J. Rajaram S. Fox E. B. (2012). Learning and remembering with others: the key role of retrieval in shaping group recall and collective memory. Soc. Cogn. 30, 121–132. doi: 10.1521/soco.2012.30.1.121, PMID: 25431516 PMC4244002

[ref8] Basden B. H. Basden D. R. Bryner S. Thomas R. L. (1997). A comparison of group and individual remembering: does collaboration disrupt retrieval strategies? J. Exp. Psychol. Learn. Mem. Cogn. 23, 1176–1189. doi: 10.1037/0278-7393.23.5.1176 9293628

[ref9] Basden B. H. Basden D. R. Henry S. (2000). Costs and benefits of collaborative remembering. Appl. Cogn. Psychol. 14, 497–507. doi: 10.1002/1099-0720(200011/12)14:6<497::aid-acp665>3.0.co;2-4

[ref10] Bouchard T. J. Hare M. (1970). Size, performance, and potential in brainstorming groups. J. Appl. Psychol. 54, 51–55. doi: 10.1037/h0028621 5415668

[ref11] Clark N. K. Stephenson G. M. (1995). Social remembering: individual and collaborative memory for social information. Eur. Rev. Soc. Psychol. 6, 127–160. doi: 10.1080/14792779443000030

[ref12] Congleton A. R. Rajaram S. (2011). The influence of learning methods on collaboration: prior repeated retrieval enhances retrieval organization, abolishes collaborative inhibition, and promotes post-collaborative memory. J. Exp. Psychol. Gen. 140, 535–551. doi: 10.1037/a0024308, PMID: 21744986

[ref13] Diehl M. Stroebe W. (1987). Productivity loss in brainstorming groups: toward the solution of a riddle. J. Pers. Soc. Psychol. 53, 497–509. doi: 10.1037/0022-3514.53.3.497

[ref14] Diehl M. Stroebe W. (1991). Productivity loss in idea-generating groups: tracking down the blocking effect. J. Pers. Soc. Psychol. 61, 392–403. doi: 10.1037/0022-3514.61.3.392

[ref15] Ekeocha J. O. (2021). Is exposure to the memories of others a necessary precondition for collaborative inhibition? Adv. Cogn. Psychol. 17, 221–229. doi: 10.5709/acp-0331-z 38169526 PMC10697717

[ref16] Finlay P. Hitch G. J. Meudell P. R. (2000). Mutual inhibition in collaborative recall: evidence for a retrieval-based account. J. Exp. Psychol. Learn. Mem. Cogn. 26, 1556–1567. doi: 10.1037/0278-7393.26.6.1556, PMID: 11185782

[ref17] Harris C. B. Barnier A. J. Sutton J. (2013). Shared encoding and the costs and benefits of collaborative recall. J. Exp. Psychol. Learn. Mem. Cogn. 39, 183–195. doi: 10.1037/a0028906, PMID: 22686851

[ref18] Harris C. B. Barnier A. J. Sutton J. Keil P. G. Dixon R. A. (2017). “Going episodic”: collaborative inhibition and facilitation when long-married couples remember together. Memory 25, 1148–1159. doi: 10.1080/09658211.2016.1274405, PMID: 28071300 PMC5503797

[ref19] Hood A. V. B. Whillock S. R. Meade M. L. Hutchison K. A. (2023). Does collaboration help or hurt recall? The answer depends on working memory capacity. J. Exp. Psychol. Learn. Mem. Cogn. 49, 350–370. doi: 10.1037/xlm0001155 36006719

[ref20] Hyman I. E. Cardwell B. A. Roy R. A. (2013). Multiple causes of collaborative inhibition in memory for categorized word lists. Memory 21, 875–890. doi: 10.1080/09658211.2013.769058, PMID: 23439153

[ref21] Jenkins J. J. (1979). “Chapter 20. Four points to remember: a tetrahedral model of memory experiments” in Levels of Processing in Human Memory. eds. Cermak L. S. Craik F. I. M. (London: Psychology Press), 429–446.

[ref22] Johansson O. Andersson J. Rönnberg J. (2000). Do elderly couples have a better prospective memory than other elderly people when they collaborate? Appl. Cogn. Psychol. 14, 121–133. doi: 10.1002/(sici)1099-0720(200003/04)14:2<121::aid-acp626>3.0.co;2-a

[ref23] Marion S. B. Thorley C. (2016). A meta-analytic review of collaborative inhibition and postcollaborative memory: testing the predictions of the retrieval strategy disruption hypothesis. Psychol. Bull. 142, 1141–1164. doi: 10.1037/bul0000071 27618544

[ref24] Meade M. L. Roediger H. L. (2009). Age differences in collaborative memory: the role of retrieval manipulations. Mem. Cogn. 37, 962–975. doi: 10.3758/mc.37.7.962, PMID: 19744936

[ref25] Meudell P. R. Hitch G. J. Boyle M. M. (1995). Collaboration in recall: do pairs of people cross-cue each other to produce new memories? Q. J. Exp. Psychol. 48, 141–152. doi: 10.1080/14640749508401381, PMID: 7754079

[ref26] Meudell P. R. Hitch G. J. Kirby P. (1992). Are two heads better than one? Experimental investigations of the social facilitation of memory. Appl. Cogn. Psychol. 6, 525–543. doi: 10.1002/acp.2350060606

[ref27] Peker M. Tekcan A. İ. (2009). The role of familiarity among group members in collaborative inhibition and social contagion. Soc. Psychol. 40, 111–118. doi: 10.1027/1864-9335.40.3.111

[ref28] Pereira-Pasarin L. P. Rajaram S. (2011). Study repetition and divided attention: effects of encoding manipulations on collaborative inhibition in group recall. Mem. Cogn. 39, 968–976. doi: 10.3758/s13421-011-0087-y, PMID: 21503804

[ref29] Reysen M. B. Bliss H. Baker M. A. (2018). Survival processing eliminates collaborative inhibition. Q. J. Exp. Psychol. 71, 1340–1347. doi: 10.1080/17470218.2017.1318408, PMID: 28398121

[ref30] Roenker D. L. Thompson C. P. Brown S. C. (1971). Comparison of measures for the estimation of clustering in free recall. Psychol. Bull. 76, 45–48. doi: 10.1037/h0031355

[ref31] Ross M. Spencer S. J. Linardatos L. Lam K. C. H. Perunovic M. (2004). Going shopping and identifying landmarks: does collaboration improve older people’s memory? Appl. Cogn. Psychol. 18, 683–696. doi: 10.1002/acp.1023

[ref32] Rossi-Arnaud C. Cestari V. Marques V. R. S. Gabrielli G. B. Spataro P. (2017). Collaboration in implicit memory: evidence from word-fragment completion and category exemplar generation. Psychol. Res. 81, 55–65. doi: 10.1007/s00426-015-0725-2, PMID: 26612200

[ref33] Saraiva M. Albuquerque P. B. Arantes J. (2016). Elimination of collaborative inhibition effect using the method of loci. Psicothema 28, 181–186. doi: 10.7334/psicothema2015.241, PMID: 27112816

[ref34] Saraiva M. Albuquerque P. B. Garrido M. V. (2023). Collaborative inhibition effect: the role of memory task and retrieval method. Psychol. Res. 87, 2548–2558. doi: 10.1007/s00426-023-01821-z, PMID: 37027039

[ref35] Stroebe W. Diehl M. (1994). Why groups are less effective than their members: on productivity losses in idea-generating groups. Eur. Rev. Soc. Psychol. 5, 271–303. doi: 10.1080/14792779543000084

[ref36] Takahashi M. Saito S. (2004). Does test delay eliminate collaborative inhibition? Memory 12, 722–731. doi: 10.1080/09658210344000521, PMID: 15724361

[ref37] Weldon M. S. Bellinger K. D. (1997). Collective memory: collaborative and individual processes in remembering. J. Exp. Psychol. Learn. Mem. Cogn. 23, 1160–1175. doi: 10.1037/0278-7393.23.5.1160, PMID: 9293627

[ref38] Weldon M. S. Blair C. Huebsch P. D. (2000). Group remembering: does social loafing underlie collaborative inhibition? J. Exp. Psychol. Learn. Mem. Cogn. 26, 1568–1577. doi: 10.1037/0278-7393.26.6.1568, PMID: 11185783

[ref39] Whillock S. R. Meade M. L. Hutchison K. A. Tsosie M. D. (2020). Collaborative inhibition in same-age and mixed-age dyads. Psychol. Aging 35, 963–973. doi: 10.1037/pag0000490, PMID: 32406708

[ref40] Wright D. B. Klumpp A. (2004). Collaborative inhibition is due to the product, not the process, of recalling in groups. Psychon. Bull. Rev. 11, 1080–1083. doi: 10.3758/bf03196740, PMID: 15875979

